# 高原红细胞增多症多组学研究进展

**DOI:** 10.3760/cma.j.cn121090-20240318-00100

**Published:** 2024-08

**Authors:** 桂萍 郑, 蔚 年, 雪峰 石, 友邦 解

**Affiliations:** 1 青海省人民医院血液风湿科，西宁 810007 Department of Hematology, Qinghai Provincial People's Hospital, Xining 810007, China; 2 青海省人民医院老年医学科，西宁 810007 Department of Geriatrics, Qinghai Provincial People's Hospital, Xining 810007, China; 3 青海省人民医院呼吸与危重症医学科，西宁 810007 Department of Respiratory and Critical Care Medicine, Qinghai Provincial People's Hospital, Xining 810007, China

## Abstract

慢性高原病（chronic mountain sickness，CMS）或Monge综合征是一种在海拔超过2 500 m地区流行的疾病，高原红细胞增多症（high altitude polycythemia，HAPC）是CMS的亚型之一。藏族人群基因组中的高原适应基因以EPAS1和EGNL1最为关键；HIF-PHD-VHL蛋白系统在HAPC发病机制中发挥重要的作用；SENP1基因编码一种蛋白酶，具有调节缺氧相关转录因子如HIF和GATA的功能，调节EPO或EPO受体表达参与红细胞生成。随着基因检测和组学技术的发展，关于HAPC发病机制的研究在转录组学、蛋白组学和代谢组学方面取得了一些新的进展，为骨髓血液生态和造血调控方面的研究奠定基础。CMS的诊断标准存在一定的局限性，尤其是高原红细胞增多症患者的诊断应该完善先天性和真性红细胞增多症推荐的基因检测。本文就HAPC在各组学技术、造血调控及诊断流程方面的最新研究作一综述，更有利于对HAPC发病机制的认识，强调基因检测在红细胞增多的临床诊断的重要性。

## Abstract (en)

Chronic mountain sickness（CMS）or Monge syndrome is a disease that is prevalent at altitude above 2 500 meters. High altitude polycythemia（HAPC）is one subtype of CMS. EPAS1 and EGNL1 are the most critical high-altitude adaptation genes in the genome of the Tibetan population. The HIF-PHD-VHL system plays an important role in the pathogenesis of HAPC. The protease encoded by the SENP1 gene regulates hypoxia related transcription factors such as HIF and GATA to affect the expression of EPO or EPOR involved in red blood cell generation. With the development of genetic testing and omics technology, new progress in the fields of metabolomics, proteomics and metabolomics has been made in the pathogenesis of HAPC. The above new research results provide a preliminary basis for bone marrow hematoecology and hematopoietic regulation of HAPC. The diagnostic criteria for CMS have certain limitations, especially in patients with excessive erythrocytosis who should undergo genetic testing recommended for congenital and polycythemia vera. This article provides a review of the latest research on HAPC in various omics techniques, hematopoietic regulation and diagnostic processes which is more conducive to understand the pathogenesis. The clinical diagnosis of excessive erythrocytosis emphasizes the importance of genetic testing.

慢性高原病（chronic mountain sickness，CMS）或Monge综合征是一种在世界海拔超过2 500 m地区流行的进行性丧失生活劳动能力综合征，全球超过1.4亿人生活在海拔2 500 m以上地区，患CMS的风险平均为5％～10％[1]。高原红细胞增多症（high altitude polycythemia，HAPC）作为CMS的一种类型，主要表现为过度的红细胞增多（excessive erythrocytosis，EE）同时符合CMS诊断标准[2]。Swenson等[3]研究发现居住高海拔地区导致红细胞比容和HGB浓度逐渐上升，EE的发生率和CMS的发病率逐渐增加，但发生EE和CMS之间大约有6年的时间。出现EE症状和CMS的具体机制目前未完全明确，也缺乏有效的干预措施。HAPC作为高原常见的CMS之一，高原是其最主要的环境因素，高原显著特征是低氧，而导致HAPC的关键是环境持续缺氧，目前HAPC的发病机制的主要研究集中在人体对高原失适应导致缺氧病理生理系统性反应，主要表现在遗传背景、表观基因组和对慢性缺氧的生理失适应反应的生命全周期的过程中[4]。本文就HAPC多组学发病机制的研究及诊断中的问题作综述，更深入的认识HAPC及其临床诊断的规范化。

一、高原适应基因组学

2010年华大基因报道显示，藏族人群对高海拔适应的自然选择的最强信号来自内皮Per-Arnt-Sim（PAS）结构域蛋白1（PAS domain-containing protein 1，EPAS1）[5]，EPAS1的单核苷酸多态性（SNP）在藏族和汉族人群之间的等位基因频率差异为78％，这是迄今为止在人类基因中观察到的最显著的等位基因频率变化，该SNP与红细胞丰度的关联性表明EPAS1在缺氧适应中发挥重要作用。2014年中外学者报道藏族人群高海拔适应的EGLN1和PPARA单倍型基因与其特有的低HGB浓度显著相关[6]，88％的藏族人携带着EGLN1基因的一个单碱基突变c.［12C>G；380G>C］，由EGLN1编码的脯氨酰羟化酶2（PHD2）触发缺氧诱导因子（HIF）的降解，PHD2 p.［Asp4Glu；Cys127Ser］突变表现出较低的氧K（m）值，在缺氧条件下促进HIF降解增加，而EGLN1 c.［12C>G；380G>C］突变消除了缺氧诱导和HIF介导的红细胞生成增加，使得藏族人群更好地适应低氧的高海拔环境。正常PHD2较PHD2 p.［Asp4Glu；Cys127Ser］突变对HIF的降解能力下降，从而导致红细胞加速生成，相反，PHD2突变增加HIF降解，红细胞生成作用降低导致适应高海拔藏族人群血液中红细胞及HGB浓度较低[7]。

一项典型的全基因组关联研究发现EPAS1、PPARA、ARNT2、THRB与低氧适应相关[8]。已有许多研究利用全基因组测序（WGS）寻找藏族人群基因组中的高原适应基因，共报道682个基因，但只有EPAS1和EGLN1两个基因能成功重复，其他基因的选择性信号仍不准确，中国学者为了全面评估藏族人群高原适应基因，绘制千人WGS构建藏族人群基因组参考图谱，样本覆盖了中国青藏高原的主要人口分布区域，发现了3 500万个变异位点，其中三分之一以上是新发现的变异位点，并利用大规模WGS数据，构建等位基因频率和连锁不平衡谱，提供了藏族人群基因组参考Panel——1KTGP[9]。此外，研究通过多信号综合分析方法重新筛选了藏族人群基因组中的高原适应基因，包括192个基因的4 320个变异位点，涉及多个生理系统和器官，支持藏族人群高原适应是多基因效应的假说，其中4个具有强选择信号的新基因（TMEM132C、ATP13A3、SANBR和KHDRBS2）与藏族人群更好的心肺功能相关[9]。参与机体黑色素合成的基因GNPAT在藏族人群中存在很强的正选择信号，位于该基因上游的一个增强子调控元件发生了点突变（rs75356281），其衍生等位基因频率在藏族人群中达58％，而在世界其他人群中的比例仅为0～18％，提示自然选择导致了此突变在藏族人群中的富集，rs75356281在藏族人群中与原肤色（臀部）和继发性肤色（手背）均存在显著的相关性[10]。上述研究提示特殊基因在藏族人群适应高原环境中发挥重要作用，能使机体保持较少的红细胞数量和较低HGB浓度，避免环境低氧引起机体缺氧。

二、HAPC的多组学研究

1. 转录组学：HAPC患者血浆外泌体miR-122-5p、miR-423-5p、miR-4433b-3p、miR-1291和miR-106b-5p表达水平显著上调，miR-200c-3p表达下调[11]，通过RNA-Seq鉴定并验证了一组长链非编码RNA（lncRNA）的功能，发现缺氧诱导的激酶介导红细胞生成调节因子（HIKER）/LICO228在CMS患者骨髓CD34^+^细胞向红细胞分化中起着关键作用，在缺氧条件下，HIKER下调CSNK2B使红细胞生成显著减少[12]。高钰琪研究团队[13]报道青藏高原移居汉族人HAPC外周血基因表达谱鉴定出5个上调、4个下调的差异表达基因，其中细胞分裂周期42（CDC42）可能促进HAPC患者骨髓红细胞生成。有学者对正常人外周血和骨髓来源的CD34^+^造血干细胞（HSC）的miRNA的表达进行分析，将miRNA靶基因预测结果和对应的mRNA表达谱进行结合，得到较为全面的人CD34^+^ HSC miRNA与mRNA相互作用的图谱[14]。但是在转录组学方面，目前HAPC的报道相较单一组学的研究而言，主要是表达谱的结果及相关的生物学信息分析，深入的机制研究较少，无法将miRNA、mRNA和lncRNA的相互作用清晰地呈现。结合以往的研究报道，HAPC患者的红细胞及血浆外泌体miR-210表达增高[15]，汉族EPAS1基因中存在HAPC的易感位点rs6756667，其与汉族男性HAPC患者发病相关，其A等位基因是汉族男性HAPC的保护因素[16]，但是位点的可重复性仍需验证。

2. 蛋白组学：目前公认最主要的是HIF-PHD-VHL蛋白系统，HIF通过调节数百个缺氧控制基因的转录来协调细胞内信号传导以应对缺氧[17]。HIF是异二聚体转录因子，由1个β亚基（HIF1β）和3个氧敏感亚基（HIF1α、HIF2α或HIF3α）组成。在常氧条件下，PHD介导的HIF脯氨酰羟基化后产生和VHL蛋白的结合位点，羟基化的HIFα与VHL蛋白结合后被E3-泛素连接酶复合物识别后降解。因此，PHD蛋白和VHL蛋白是HIF的负调控因子，编码它们的基因的功能缺失或突变会损害HIFα的泛素化和降解，导致HIFα积累[18]。在缺氧条件下，PHD酶活性降低导致HIFα积累，与HIF1β形成异二聚体复合物，随后转移到细胞核内与辅因子CBP/p300和Pol Ⅱ复合物等相互作用，并与DNA中的缺氧反应元件结合，启动或增强HIF依赖性基因的转录。在秘鲁塞罗德帕斯科山城（秘鲁的一个小镇，海拔4 340 m）生活的健康高地人和CMS患者中发现，EE患者的血浆可溶性EPO受体（sEPOR）水平下降，HGB浓度随着EPO/sEPOR的增加呈指数级上升，EE与更高的EPO/sEPOR值密切相关，导致血浆EPO结合膜EPO受体（mEPOR）的可用性升高，从而对红细胞生成产生更强的刺激[19]。SENP1基因编码一种蛋白酶，具有调节缺氧相关转录因子如HIF和GATA的功能，调节EPO或EPOR表达参与红细胞生成[20]。CMS患者的造血祖细胞在缺氧条件下产生更高比例的红系前体细胞，而SENP1对造血祖细胞的增殖至关重要[21]。此外，有研究提示HAPC相关肾脏疾病的病理变化包括肾小球和肾小球外血管病变，肾脏对EPO内分泌功能以及骨髓红系祖细胞mEPOR表达的调控也许在红细胞的生成中同样发挥极其重要的作用[22]。高原地区EE患者普遍存在VHL基因拷贝数下降，EPO水平升高[23]，而HAPC患者EPO水平正常或增加，且VEGF水平在骨髓及外周血均增加[24]–[25]。

3. 代谢组学：CMS模型大鼠血液中的多种氨基酸水平升高，β-葡萄糖和α-葡萄糖水平也显著升高[26]。红细胞缺氧代谢重编程在高海拔地区正常人和机体易缺氧的患者（如镰刀型红细胞病和慢性肾病患者）中发挥重要的作用，可溶性CD73升高致循环腺苷增加，激活A2B腺苷受体（adenosine A2B receptor，ADORA2B），通过红细胞特异性合成1-磷酸鞘氨醇（sphingosine-1-phosphate，S1P）协同促进2,3-二磷酸甘油酸盐（2,3-biphosphoglycerate，2,3-BPG）的产生调节氧离曲线，随后发现红细胞平衡核苷转运蛋白1（equilibrative nucleoside transporter 1，eENT1）是控制高海拔人类和缺氧小鼠血浆腺苷水平的关键因子，缺氧诱导eENT1降解从而加速血浆腺苷升高[27]。在海拔4 300 m、氧含量为14.07％的高海拔缺氧环境中饲养的大鼠体重和内脏脂肪组织重量显著降低，甘油二酯和甘油三酯的浓度也显著降低[28]。富氧180 d后HGB恢复正常的HAPC患者，其体内戊糖磷酸途径（pentose phosphate pathway，PPP）增加[29]，NADP还原为NADPH，NADPH可调节谷胱甘肽进而保护组织免受活性氧的侵害、修复红细胞中携氧的氧化蛋白、促进促氧化剂和抗氧化剂之间的再平衡[30]，但是在未恢复患者中检测到PPP减少，表明PPP可能参与缺氧诱导的红细胞增多症的进展。有学者已经证明缺氧会阻碍红细胞代谢向PPP的转移[31]，缺氧可能导致线粒体和内质网功能障碍，过度激活NADPH氧化酶、黄嘌呤氧化酶和一氧化氮合酶解偶联，从而形成氧化应激反应[32]–[33]。超高海拔地区中青年藏族男性中高尿酸血症的患病率及其相关因素分析显示，49.4％患有高尿酸血症，高尿酸血症的风险与体重指数、甘油三酯、红细胞计数和肌酐水平呈正相关，与糖尿病的存在呈负相关，其中红细胞增多症患者的高尿酸血症患病率显著高于非红细胞增多症患者，尿酸水平与红细胞压积和HGB水平呈正相关[34]。上述研究提示HAPC相关的代谢紊乱可影响氧离曲线、糖代谢、脂肪代谢以及嘌呤代谢，从而引起机体如红细胞增多、低氧血症、血脂异常、高尿酸血症等异常，尚无相关药物研究逆转上述代谢的变化。目前比较一致的是HAPC患者ADORA2B基因多态性影响红细胞的氧离曲线[35]，ADORA2B基因SNP位点rs2015353的A等位基因是中国汉族人群HAPC的危险因素，ADORA2B基因SNP位点rs2779212的C等位基因是中国汉族人群HAPC的保护因素[36]，但因目前文献报道较少，ADORA2B基因SNP位点的可重复性仍需验证。

三、骨髓造血微环境及红系造血调控

成熟红细胞的数量与骨髓红系细胞的增殖、分化密切相关，骨髓红系细胞通过与造血微环境的相互作用维持机体内红细胞的稳态，过程涉及红系细胞外的调节因子、细胞内的遗传学和表观遗传学调控等多个环节[38]。造血分化是指由HSC定向分化成为各系祖细胞，再继续分化发育成多种具有不同形态和功能的成熟血细胞的过程。造血分化是一个极其复杂的调控过程，既涉及到表观遗传学和转录水平的调控，又有转录后水平、翻译和翻译后水平的调控[37]。随着红系细胞的分化，许多红细胞膜表面抗原的表达随着时间的推移而变化，可以通过流式细胞术追踪和定量分化。基于CD34/CD36/CD71和CD105在CD123（IL-3受体）阴性细胞中的顺序表达，可以富集红系祖细胞的不同亚群。结合GYPA（CD235A）和CD105或者band 3和α4整合素的表达，可以跟踪红系细胞成熟过程[38]。尽管这些表面标志物能够富集这种分化过程的不同阶段，但分类的细胞群仍然是异质的，而一些具有中间抗原表达的亚群可能很难确定分类，新兴的单细胞基因组研究表明细胞状态的连续性有助于定义具有不同功能特性的特定红系亚群，体现不同阶段内和阶段之间的异质性和实质性重叠[39]–[40]。红系分化中转录因子GATA-2在红系前体细胞中高表达以维持细胞干性，而随着GATA-1的逐渐增强，推进着红系前体细胞向成熟红细胞分化[41]–[43]，到分化末期有EKLF、NF-E2等转录因子参与[44]。EPO是在成年期间于肾脏中产生，协同造血生长因子（HGF），刺激骨髓红细胞生成。缺氧通过氧敏感反馈回路激活EPO的分泌，EPO通过与EPO-R结合增加细胞存活率，将祖细胞红系爆式集落形成单位（Burst-forming unit-erythroid，BFU-E）和红系克隆形成单位（colony-forming unit-erythroid，CFU-E）驱动成熟为数十亿个成熟的红细胞。该途径涉及多种红系转录因子的激活，如GATA1、FOG1、TAL-1、EKLF和BCL11A，并导致编码参与血红素生物合成和HGB产生的酶的基因过表达。除骨髓红系祖细胞之外，大脑、心脏和骨骼肌中检测到EPO-R的异二聚体复合物，这体现了EPO的多效性，包括诱导多能间充质干细胞（MSC）以及调节先天免疫和适应性免疫细胞，如EPO可通过激活EPOR信号通路诱导多能MSC的成骨和内皮转化，导致骨重塑、诱导血管生成和大量营养因子的分泌[45]。正常红细胞生成的主要调控机制包括外部造血细胞因子、造血细胞因子受体、转录因子和信号分子[37],[46]，但在HAPC的发病机制中占主导地位的关键基因或蛋白仍未明确。

四、低氧相关敏感通路和HAPC红系造血调控

CMS患者骨髓细胞中的HIF-2α、EPO的mRNA和蛋白表达均增加，HIF-2α的表达和EPO、HGB水平相关，且骨髓微血管密度和HGB相关，骨髓细胞通过HIF-2α/EPO通路影响骨髓龛自分泌和（或）旁分泌机制来调节红细胞和血管的生成[47]–[48]。蛋白质组学分析发现CMS患者血浆中HGB β链、硫氧还蛋白-1和磷酸甘油酸激酶1有助于CMS的诊断[49]。在CMS模型大鼠中观察到慢性缺氧显著诱导VHL启动子CpG位点的甲基化，通过降低VHL的表达而上调HIF-2α/EPO通路，从而导致红细胞增多[50]。HIF-2α缺陷的小鼠的EPO表达未受影响，但出现红细胞成熟障碍和贫血，这是由于HIF-1α可以结合到由HIF-2α调控基因的HRE区域补偿恢复HIF-2α一些靶基因的表达[51]。PHD作为氧传感器，在抑制PHD时可以激活缺氧反应，而在常氧环境无此效应，基于抑制PHD的小化合物通过增加HIF-1的积累而提高EPO的分泌的理论机制，PHD抑制剂在治疗肾性贫血方面已经广泛应用[52]。总之，肾脏EPO的分泌调节功能和骨髓红系造血之间有着密切的联系，有研究报道藏族HAPC患者肾活检提示有肾小球和肾小球外血管病变，同时HIF-2α在肾组织中高表达[22]。骨髓微环境对造血细胞的调控发挥重要的作用，如支持造血及调节造血细胞定居、增殖、分化和发育等。HAPC红细胞过多可能是骨髓微环境红系调控及骨髓外低氧因素共同影响的结果。了解HAPC患者骨髓红系干祖细胞的生物学特征、基因表达以及红系造血相关转录因子之间的联系、红系造血和免疫细胞之间的微调控机制以及筛选对HAPC红系造血调控关键的途径或关键蛋白，可能为干预HAPC或预防提供更好的策略。

五、HAPC的精准诊断及意义

HAPC作为CMS的亚型，主要有EE的症状同时符合CMS诊断标准[2]，但是目前诊断标准没有明确排除血液系统疾病所致的红细胞增多，主要包括原发性先天性家族红细胞增多症（FE）和继发性先天性红细胞增多症。有文献报道原发性先天性FE和EPOR的突变有关[53]，EPOR突变是原发性1型FE（ECYT1）的分子指标，也称原发性先天性FE。继发性先天性红细胞增多症是由于红细胞生成促进物质作用于造血祖细胞的异常调节引起的，其特点是由于氧感应通路缺陷（ECYT2-5）或影响HGB氧亲和力的变异体（ECYT6-8）导致血清EPO水平正常或偏高[54]，主要和HBB、BPGM、VHL、EGLN1和EPAS1突变相关[55]。而原发性红细胞增多症红细胞数量增加的主要原因是真性红细胞增多症（PV）（获得性）或原发性先天性FE，其中JAK2基因突变是PV的分子学指标，故在临床中红细胞增多症的诊断遵循上述血液系统基因突变所致的各种类型作为参考[56]，在诊断HAPC时，完善原发性、继发性和PV推荐的基因检测，识别出符合诊断为HAPC患者中属于上述两种原因的患者，有利于干预治疗。

六、总结

EGLN1和EPAS-1基因在高海拔低氧适应和失适应中发挥重要的作用，而在HAPC发病中骨髓红系造血微环境调控和肾脏EPO-骨髓红系EPO途径在红细胞生成中的作用大小，及低氧相关的关键蛋白尚未明确。上述的研究报道藏族人群基因组数据具有样本量小、低变异密度、检测方法单一等局限性，并且无论是遗传适应、转录组学、代谢组学、表观遗传学还是造血微环境方面，均为单一方法学去分析HAPC的发病机制，仍无法解释HAPC的发病机制的核心，无法整合为整体生命活动的机体中分析相互作用的机制。未来需应用多组学多手段整合到同一群体，挖掘环境缺氧导致机体出现病理生理反应的主要通路和关键蛋白，了解骨髓血液生态，从而为预防和治疗提供更有说服力的参考依据。同时对符合HAPC诊断标准的患者进行血液遗传和肿瘤全外显子测序、识别先天性和PV患者、制定HAPC诊断流程，从而优化诊断标准对HAPC的诊治有很大的临床意义。
